# Treatment of Children with Advanced-Stage Lymphoblastic Lymphoma with Pegaspargase

**Published:** 2014-01-26

**Authors:** Zhang Yu-tong, FENG Li-hua, Zhong Xiao-dan, Wang Li-zhe, Chang Jian

**Affiliations:** 1 Department of Pediatric Hematology and Oncology; 2 Department of Obstetrics and Gynecology, the First Hospital, Jilin University, Changchun, Jilin, China

**Keywords:** Pegaspargase, L-Asparaginase, Lymphoblastic Lymphoma, Chemotherapy

## Abstract

***Objective:*** To evaluate the feasibility of Pegaspargase instead of L-asparaginase to treat children with advanced-stage lymphoblastic lymphoma (LBL) on the Berlin-Frankfurt-Munster (BFM)-95 protocol.

***Methods:*** Fifty-four newly diagnosed patients with stage III or IV LBL and without any treatment were enrolled in this study. Pegaspargase took place of L-asparaginase in BFM-95. The complications and treatment responses of patients treated on the BFM-95 protocol and modified BFM-95 protocol were then evaluated respectively.

***Findings*** ***:*** For LBL patients treated with BFM-95 protocol or modified BFM-95 protocol, the complete response, event-free survival, overall survival were similar. Stage 4 myelosuppression was the most common complication in both groups. Besides that, among 31 patients receiving modified BFM-95 protocol, coagulation defects were the most common complication. In contrast, anaphylactic reaction was the most common complication in the other 23 patients receiving BFM-95 protocol.

***Conclusion:*** Modified BFM-95 protocol is available to children with advanced-stage LBL with an equal outcome and enhances its compliance and decreases the incidence of anaphylactic reaction, compared to BFM-95 protocol. Coagulation defects are the major complication and tolerable in modified one.

## Introduction

Lymphoblastic lymphoma (LBL) is a highly malignant tumor of variegated lymphoid reticuloendothelial cells, one common histologic subtype of non-Hodgkin’s lymphoma in childhood^[^^1^^]^. It is clear that along with the development of multimodality therapy, the prognosis of children with LBL have been markedly improved, where L-asparaginase is an important and universal component in chemotherapy^[^^2^^,^^3^^]^. It can significantly improve long-term event-free survival^[^^4^^,^^5^^]^. However, asparaginases are associated with a unique set of side effects^[^^3^^]^. Generally, asparaginase is cleared rapidly with an apparent half life of about 20 hours^[^^6^^]^. In previous clinical trials, the drug had to be administered intramuscularly 6-10 times every other day for maintaining its effective drug activity. Moreover, clinical hypersensitivity reactions and silent inactivation due to antibodies against E. coli-asparaginase, led to inactivation of E. coli-asparaginase in up to 60% of cases^[^^3^^,^^7^^]^. Polyethylene glycosylated–asparaginase (pegas-pargase), formed by covalently attaching polyethylene glycol to the native *Escherichia coli* enzyme, was developed for reducing the immunogenic potential. After one single dose of this drug, the potential therapeutic enzyme activity can be maintained for at least 2 weeks^[^^8^^,^^9^^]^. For these reasons, we explored to use pegaspargase instead of L-asparaginase to treat children with advanced-stage LBL in order to improve patient’s treatment compliance and reduce the risk of acute anaphylactic reaction.

## Subjects and Methods

**Patients**

Between 2000 and 2006, children younger than 16 years who had a previously untreated LBL on admission to our hospital with advanced features, defined as stage Ⅲ and stage Ⅳ diseases on the St Jude staging system, were eligible for the trial. The Institutional Review Boards approved the protocol before enrollment. Written informed consents were obtained from their parents or legal guardians before starting therapy based on the BFM-95 protocol.

**Pretreatment evaluation of stage and diagnosis**

The initial diagnostic workup for LBL included a detailed physical examination and bone marrow aspiration, computed tomography (CT) scan of the chest and abdomen, bone scintigraphy, immunophenotyping study and examination of the cerebrospinal fluid (CSF). Patients were classified according to the St Jude staging system^[^^10^^]^.

**Study Design**

All patients were randomly assigned to receive treatment with either BFM-95 protocol or modified BFM-95 protocol. In modified BFM-95 protocol L-asparaginase was replaced by pegaspargase, administered by intramuscular injection (2.500 IU/m^2^/d) on days 12, 28 in induction and on day 8 in reinduction phase, which replaced 8 doses of L-asparaginase (10000 U/m^2^/d) on days 12, 15, 18, 21, 24, 27, 30 and 33 in induction and 4 doses on days 8, 11, 15 and 18 in reinduction, respectively, as defined by the Non-Hodgkin Lymphoma– Berlin-Frankfurt-Munster-95 (NHL-BFM-95) protocol^[^^2^^]^. No single injection dose was more than 2.0 mL (1500 IU) at one injection site. The doses and administration modes of the other chemotherapeutic agents were the same as in BFM-95 protocol.

**Response definitions and toxicity assessment**

Complete response (CR) was defined as no evidence of tumor by physical examination and imaging studies (CT scans or magnetic resonance imaging). Bone marrow aspirate was needed. Patients were considered in partial response (PR) if there was a decrease of 50% or more in all measurable mass lesions. No response (NR) was defined as less than 50% reduction in the extent of disease^[^^11^^]^. Event-free survival (EFS) was defined as the interval between diagnosis and disease progression, relapse, or death; and overall survival (OS) was defined as the interval between diagnosis and death from any cause or last contact. During treatment, patients were closely monitored on peripheral blood cell counts, major organ functions (liver, renal and cardiac functions), serum electrolytes, and coagulation functions, etc. All toxicity were collected and graded based on the National Cancer Institute Common Terminology Criteria for Adverse Events, version 4.0^[^^12^^]^.

 After completion of chemotherapy, patients entered the follow-up and received the periodic reevaluation on biochemical and imaging studies.

**Statistical Design and Analysis**

The primary outcome measures for this study were EFS and OS. Life-table estimates of survival time were calculated by the method of Kaplan and Meier for outcome comparison. The chi-square test was used to analyze significance between the two groups, comparing the treatment toxicity. Outcome was assigned to the randomized treatment, regardless of the therapy received. *P*<0.05 was considered significant.

## Finding

**Patients**

Between June 2000 and December 2006, 57 patients were assigned to this study. 

**Table 1:** Clinical characteristics of the 54 patients at presentation

**Variable**	**Characteristic**	** No Patients (%)**
**Sex**	**Female**	19 (35.2)
	**Male**	35 (64.8)
**Immunophenotyping**	**T-lineage**	40 (74.1)
	**B-lineage**	14 (25.9)
**Stage category**	**stage ** **Ⅲ**	30 (55.6)
	**stage ** **Ⅳ**	24 (44.4)
**Chemotherapy**	**NHL-BFM-95 protocol**	23 (42.6)
	**modified NHL-BFM-95 protocol**	31 (57.4)

NHL-BFM-95 protocol: Non-Hodgkin Lymphoma– Berlin-Frankfurt-Munster-95 protocol

Three patients were excluded because lack of complete documentation. Mean age of the remaining 54 patients was 5.1±2.58 (95%CI, 4.40 to 5.80) years, ranging from 2.5 years to 14.7 years. On immunophenotyping study, the tumor cells were classified as T-lineage in 40 patients and B-lineage in 14 patients. 17 stage Ⅲ patients and 6 stage Ⅳ patients were assigned to BFM-95 group (B-lineage 5, T-lineage 18), while 13 stage Ⅲ patients and 18 stage Ⅳ patients were assigned to modified BFM-95 group (B-lineage 9, T-lineage 22). There was no significant difference on histologic subtypes (*P*>0.05). The clinical detailed characteristics of the remaining 54 patients are listed in Table 1.

**Treatment outcomes**

Patients’ treatment responses were evaluated on day 33 of induction and at the end of chemotherapy^[^^13^^]^. The CR rate was 97% in the modified BFM-95 group and 94% in the BFM-95 group on day 33 of induction. The 5-year overall survivals were 88%±8% (95% CI, 72.31% to 103.68%) with BFM-95 versus 91%±6% (95%CI, 79.24% to 102.76%) with modified BFM-95 (Fig 1), and the event-free survival rates were 80%±9% (95%CI, 62.36% to 97.64%) with BFM-95 versus 87±7%(95%CI, 73.28% to 100.72%) with modified BFM-95 (Fig 2)(median follow-up time, 5.75±1.33 years) (95%CI, 5.38 to 6.12) (Table 2).

**Patients with recurrence or progressive disease**

A total of 8 randomly assigned patients had a documented relapse or disease progression: 4 (9%) in the BFM-95 group and 4 (10%) in the modified BFM-95 group. Two patients in the BFM-95 group and three in the modified BFM-95 group died. Five deaths in both groups were due to disease relapse or progression. Three remaining relapsed patients, two with pre-T LBL and one with pre-B LBL, were alive. 

**Toxicity**

Among 31 patients receiving Pegaspargase during induction and reinduction phases, stage 4 myelosuppression was the most common complication. Besides, one experienced grade 3, the other four grade 2 coagulation defects. The patient with grade 3 coagulation defects had continuous capillary hemorrhage at the injection spot and prolonged APTT, the plasma FIX was lower than 30% of the normal values and the fibrinogen level was decreased to 1.0 g/L. The other four patients developing grade 2 coagulation defects had decreased fibrinogen levels which was lowered to 1.0 g/L, without any clinical symptoms for about two weeks. One developed grade 3 anaphylactic reaction (symptomatic broncho-spasm and urticaria). In addition, there was another grade 2 complication in one patient, who developed mild pancreatitis without any symptom.

**Table 2:** Treatment outcomes according to stage and randomized treatment regimen

**Regimen **	**5-year** **Event-free survival**	**5-year overall ** **survival**	**Complete ** **response**
**NHL-BFM-95**	**83%**	**91%**	**94%**
**Modified NHL-BFM-95**	**87%**	**90%**	**97%**

NHL-BFM-95 protocol: Non-Hodgkin Lymphoma– Berlin-Frankfurt-Munster-95 protocol;

**Table 3:** Toxicity differences between the two protocols

	**Modified-BFM-95**			**BFM-95**				***P *** **value**
**Total**	31				23			
**Stage 4 myelosupression**		31	100%			23	100%	
**Coagulation defects**	Grade 2	4	12.9%	Grade 2		2	8.7%	0.627
	Grade 3	1	3.2%	Grade 3		-	-	0.385
**Anaphylactic reaction**	Grade 3	1	3.2%	Grade 3		5	21.7%	0.032
	Grade 4	-	-	Grade 4		1	4.3%	0.241
**Pancreatitis**	**Grade 2**	**2**	**6.5%**	**Grade 2**		**2**	** 8.7%**	**0.756**

Increased pancreatic amylases were only found on routine screening. All of them achieved complete recovery after treatment. No hemorrhage or thrombosis was seen. There was no severe complication during the sequential phases. 

 Among other 23 patients receiving BFM-95 protocol, beside the myelosuppression, complication of anaphylactic reaction was noted. Five of 23 patients experienced grade 3 anaphylactic reaction (urticarial lesions covering larger than 30% of body surface) during reinduction, including one experiencing a severe anaphylactic shock. Two experienced grade 2 pancreatitis without any symptoms. Two developed grade 2 coagulation defects, whose fibrinogen level was decreased to 1.2 g/L for 3-5 days without prolonged PT or APTT. There is no significant difference regarding coagulation defects of the two groups (*P*<0.05). The detailed data are shown in Table 3.

## Discussion

Until now, most studies were associated with evaluating Pegaspargase on treating children with ALL, rather than lymphoma. As the common regimen for LBL is similar to leukemia’s, we attempted to use Pegaspargase to treat children with advanced-stage LBL, and the outcome showed that the response to modified BFM-95 protocol was similar to the previously reported^[^^14^^,^^15^^]^. To our knowledge, this is the first trial to treat LBL children with Pegaspargase. The result of this study indicated that Pegasparagse can be administered safely to children in combination with other chemotherapeutic agents. For both groups, stage 4 myelosuppression was the most common complication. However, it was considered to be mostly associated with the myelosuppressive activity of the other cytotoxic chemotherapy drugs. 

 Of the 31 patients treated with Pegaspargase, only one experienced a grade 3 coagulation disorder, APTT was prolonged and the patient had continuous capillary hemorrhage at the injection spot. The study of coagulation factors revealed that the plasma FIX was lower than 30% of the normal values and the fibrinogen level was decreased to 1.0 g/L. Interestingly, after administering prothrombin complex concentrate (PCC) (20 IU/kg/d), vitamin K_1_ (5 mg/d), and fibrinogen (0.5g/d), APTT was still prolonged and getting worse and worse until plasma infusion was done. This indicated that coagulation disorder in patients receiving Pegaspargase was more sensitive to response to plasma infusion rather than administering PCC, which meant Pegaspargase might influence other coagulation factors such as FII and FVII. 

 As we know, asparaginase preparations interfere with hepatic production of both coagulant and anti-coagulant proteins. Rytting reported that the incidence of thrombosis was generally 10% in pediatric patients^[^^16^^]^. Pancreatitis and thrombotic complications are less common in children than in adolescents and adults. In our study, no thrombosis was noted either in modified BFM-95 group or BFM-95 group. The incidences of other complications were similar to previously reported^[^^11^^,^^16^^]^. The major adverse reaction noted in the L-asparaginase group in our study was anaphylactic reaction. Because it is a bacterially derived protein, L-asparaginase can often induce anaphylaxis and immune responses which may lead to development of specific antibody, resulting in rapid elimination of enzyme and losing pharmacological activity. Development of the specific antibody of L-asparaginase is common, it was reported in over 50% of patients treated with multiple administrations of L-asparaginase^[^^17^^,^^18^^]^. 

 Anaphylactic reaction in the Pegasparagse group compared to L-asparaginase group is quite rare. Only 1 patient suffered from it. It was in accordance with some other studies in which Pegaspargase had a relatively lower immunogenicity due to the covalent conjugation to monomethoxy polythlene glycol and used to replace L-asparaginase in patients who had developed allergic reaction. In adults with newly diagnosed acute lymphoblastic leukemia, incidence of allergic reaction to Pegasparagse was strikingly lower compared to L-asparaginase, ranging from 0～15%^[^^4^^,^^9^^,^^16^^,^^15^^,^^19^^]^, in pediatric patients it was also lower, around 9%^[^^20^^]^. In our study, an interesting thing was that all anaphylactic reactions were observed during induction with Pegasparagse, and no such a reaction was seen in reinduction. It was in contrast to L-asparaginase. The frequency of complications would increase with continued L-asparaginase treatment. Maybe, Pegasparagse could easily cause immune tolerance because of the less frequency of administration. On the number of observed patients, we could not rule out the possibility of coincidence. Further clinical observation is needed.

 Recently, Children’s Oncology Group applied Erwinia asparaginase as an alternative drug in case of hypersensitivity to Pegaspargase, the outcome was excellent^[^^21^^]^. However, Erwinia asparaginase is not commercially available now. 

 In CALGB 9511 clinical trial, pegaspargase was used in lieu of the native enzyme, the aim was to compare the differences on overall survival and disease-free survival between patients who did and did not achieve asparagine depletion. They concluded that effective asparagine depletion with Pegaspargase was feasible as part of an intensive multiagent therapeutic regimen in adult ALL and appeared associated with improved outcomes^[^^16^^]^. Our study demonstrated that the antitumor activity of Pegaspargase is comparable to L-asparaginase. Neither the EFS nor OS was significantly different between the groups. Moreover, we observed that coagulation disorder was the most common complication in patients treated with modified BFM-95 protocol (there was no significant difference between the 2 groups, *P*>0.05), while anaphylactic reaction was the major complication in patients treated with BFM-95 protocol (there was significant difference between them, *P*<0.05). In all of 31 patients who received Pegaspargase, although a few patients experienced some complications, it was well tolerated and the patients recovered soon after associated symptomatic treatment.

## Conclusion

Obviouslly, our study showed that L-asparaginase could be replaced by Pegaspargase safely to treat children with advanced-stage LBL with enhancing patient’s compliance to chemotherapy and without decreasing the OS and EFS. Even though our clinical trial was a small sample clinical trial, it can indicate the benefits of Pegaspargase. Further study is needed to evaluate the long-term outcome of Pegaspargase.
